# Maternal administration of probiotics promotes brain development and protects offspring’s brain from postnatal inflammatory insults in C57/BL6J mice

**DOI:** 10.1038/s41598-020-65180-0

**Published:** 2020-05-18

**Authors:** Jing Lu, Lei Lu, Yueyue Yu, Jillian Baranowski, Erika C. Claud

**Affiliations:** 1 The University of Chicago, Pritzker School of Medicine, Department of Pediatrics, Chicago, IL 60637 USA; 2 The University of Chicago, Pritzker School of Medicine, Chicago, IL 60637 USA

**Keywords:** Blood-brain barrier, Neuroimmunology

## Abstract

Neonatal morbidities are associated with long term neurological deficits in life and have also been associated with dysbiosis. We tested whether optimizing the neonate’s microbiome through maternal probiotic supplementation can improve offspring’s neurodevelopmental outcomes. Maternal LB supplementation, carried out by giving *Lactobacillus acidophilus* and *Bifidobacterium infantis* (LB) to pregnant C57/BL6J mice daily from E16 to weaning, significantly suppressed postnatal peripheral proinflammatory insult-induced systemic inflammation and normalized compromised blood-brain barrier permeability and tight junction protein expression in the offspring at pre-weaned age. Maternal LB exposure also regulated markers associated with leukocyte transendothelial migration, extracellular matrix injury and neuroinflammation. The suppressed neuroinflammation by maternal LB supplementation was associated with reduced astrocyte/microglia activation and downregulation of the transcriptional regulators CEBPD and IκBα. Furthermore, maternal LB supplementation promoted neuronal and oligodendrocyte progenitor cell development. Our study demonstrates the efficacy of maternal LB supplementation in modulating systemic and central nervous system inflammation as well as promoting neural/oligodendrocyte progenitor development in the offspring. This evidence suggests that maternal probiotic supplementation may be a safe and effective strategy to improve neurological outcomes in the offspring.

## Introduction

The rate of preterm birth and the cost of neurodevelopmental diseases associated with preterm birth have been rising^1,2^. Thus, there is an increasing demand to develop interventions to reduce prematurity-associated neurological deficits. Preterm infants are at high risk for neurodevelopmental complications such as periventricular leukomalacia^3^, cerebral palsy^4^, and reduced cognitive performance later in life^5^. Microbiota is defined as the collection of microbial organisms that inhabits a certain environment^6^ and dysbiosis of the gut has been associated with the onset of neurodevelopmental disorders including attention-deficit/hyperactivity disorder^5,7,8^ and autism spectrum disorders^9–11^. Dysbiosis in preterm infants also contributes to neonatal sepsis^12^, necrotizing enterocolitis (NEC)^13^ and neuroinflammation^14^, which are also associated with long term neurological deficits^15–17^.

In animal models, sub-optimal microbial communities result in neuroinflammation and are associated with delayed brain development^18–21^. In particular, we have previously shown in a transfaunation of pregnant germ-free (GF) dam model that a microbial community associated with growth retardation in human preterm infants delayed neuronal development and resulted in both systemic and central nervous system (CNS) inflammation in the offspring^20,22^. Other studies have suggested that systemic inflammation is the driving factor to trigger CNS inflammation^23,24^ and several neurological conditions including sickness behaviors, depression and impaired learning and memory, due to the contribution of circulating cytokines to the communication between the immune system and the CNS^23,25–29^.

In recent years, targeting the microbiome for host benefits has gained significant attention^30–32^. The microbiome of neonates can be affected by postmenstrual age, mode of delivery, maternal and postnatal antibiotic usage, feeding patterns, and hospital and post-discharge environments^33^. Until recently, most of the current knowledge regarding targeting the microbiome to improve neonatal outcomes came from studies investigating direct administration of probiotics, most commonly *Lactobacillus acidophilus* and *Bifidobacterium infantis* (LB), to preterm infants to prevent NEC and/or associated mortality^34–37^. Probiotics are described as “live microorganisms which when administered in adequate amounts confer a benefit to the host”^38^. Studies have strongly documented the beneficial attributes of probiotics in host physiology including regulation of pathogenic bacterial colonization, mucosal barrier integrity, mucosal IgA responses, and anti-inflammatory cytokines. However, even with emerging evidence for a microbiome-brain communication pathway, few studies have explored optimization of the neonatal microbiome as a potential therapeutic intervention to improve neurological outcomes. This is potentially due to 1) the functional down-regulation of neonatal leukocytes (e.g., neutrophils, monocytes, and NK cells) and the complement system of the innate immune system in both term and preterm infants leading to suspected higher susceptibility of neonates to infections and other pathological conditions^39^ and 2) reported sepsis cases when probiotics were given prophylactically to reduce the incidence of NEC and mortality in preterm infants^37,40,41^. Therefore, one potential alternative yet to be explored is to change the maternal microbiome to improve neurological outcomes in the offspring.

Probiotic supplementation during pregnancy is generally regarded as safe since mothers do not have the same immune system immaturities as the neonates and has been found to confer benefit to the mother, protecting against preeclampsia^42^, gestational diabetes^43^, and vaginal infection^44^. In addition, maternal supplementation with probiotics during pregnancy and/or during lactation has been demonstrated to be an effective route to alter the infant microbiome^45,46^ as well as provide protection against diseases^47–49^. In a double-blinded placebo-controlled randomized clinical trial (RCT)^45^, antibiotics and birth mode (caesarean section) were associated with decreased *Bifidobacterium* abundance in infants. Maternal supplementation during pregnancy and breastfeeding of *Bifidobacterium breve* Bb99, *Propionibacterium freundenreichii* subsp. *shermanii* JS, *Lactobacillus rhamnosus* Lc705, and *Lactobacillus rhamnosus* GG normalized the *Bifidobacterium* abundance in the infants at three months of age. In another double-blinded placebo-controlled RCT study, both pre- and post-natal supplementation of a probiotic cocktail that included *Bifidobacterium breve* Bb99, *Lactobacillus rhamnosus* Lc705, and *Lactobacillus rhamnosus* GG reduced the risk of allergic disease among caesarean-born infants^49^. These limited but timely studies suggest that maternal probiotic supplementation can confer beneficial traits to the offspring.

In adults, probiotics have been shown to reduce circulating levels of systemic pro-inflammatory biomarkers in patients with a range of systemic inflammatory conditions including ulcerative colitis and psoriasis^50^, rheumatoid arthritis^51,52^, and liver disease^53,54^. Furthermore, a probiotic mixture (VSL#3, which contains four strains of Lactobacillus, three strains of Bifidobacterium and one Streptococcus salivarius subsp. thermophilus) has been shown to be able to reduce peripheral TNF-activated neuroinflammation marked by microglial activation and cerebral monocyte infiltration and altered sickness behaviors in the setting of peripheral organ inflammation^55^. These studies suggest that probiotics might exert effects on the CNS through an anti-inflammatory mechanism.

Therefore, we hypothesized that maternal probiotic supplementation confers protection on the CNS of offspring from inflammatory stimuli. Since IL-1β is a master regulator of neuroinflammation and elicits greater neuroinflammation when compared to other cytokines such as TNF or lipopolysaccharide (LPS, which represents exclusively gram-negative bacteria-induced inflammation)^24^, we chose to use IL-1β as the postnatal proinflammatory insult in this study. Prior to 21 days of life (weaning age) is a stage during which the rodent brain undergoes most of its neurogenesis, gliogenesis and myelination and is comparable to human infant neurodevelopment from birth to two to three years old^56^. Since studies have suggested that pre-wean rodents are more susceptible to inflammatory insults with adverse brain outcomes^57^, we investigated the effect of postnatal inflammatory insult on the offspring at pre-wean (two weeks) and post-wean (four weeks) age. The overall aim of this study was to investigate the effects of maternally administrated LB on inflammatory responses, neuroinflammation and neurodevelopment in the offspring. We demonstrate that maternally administrated LB from pregnancy to weaning protects the offspring brain from postnatal systemic proinflammatory insults and suppresses systemic inflammation-induced blood-brain barrier (BBB) dysfunction as well as immune cell activation and neuroinflammation. LB also actively promotes the development of neurons and oligodendrocyte progenitor cells in the brain.

## Results

### Maternal administration of *Lactobacillus acidophilus* and *Bifidobacterium infantis* (LB) significantly attenuated postnatal IL-1β-induced systemic inflammation in the offspring

We modeled systemic inflammation in C57BL/6J specific-pathogen free (SPF) mice at both two and four weeks of age with a peripheral immune challenge of intraperitoneal (i. p.) injection of IL-1β. As shown in Table 1, in two-week old mice four hours after injection, the serum levels of IL-6, KC, MCP-1, and IL-1β, but not IL-1α, evaluated by multiplex ELISA were significantly higher than those with saline injections (at least *p* < 0.01, n = 3). At four weeks of life there was not a significant inflammatory cytokine response to IL-1β injection (Table 2) (*p* > 0.05, n = 3) except for an increase in the serum MCP-1 level (*p* < 0.05, n = 3).

**Table 1:** Systemic cytokine/chemokine levels after four hours i.p. IL-1β injection in the offspring at two-week old age.

mean±S.E.M (pg/mL) Treatment	IL-6	KC	MCP-1	IL-1α	IL-1β
Saline	24 ± 23	128.8.5 ± 94.51	36.32 ± 29.94	89.82 ± 9.45	6.49 ± 5.75
IL-1β (i.p.)	18747 ± 6037*	20339 ± 386.5*	13195 ± 1273*	91.66 ± 12.25	1019 ± 215.3*

Results are presented as: mean ± S.E.M, n = 3. *Comparison was performed with t-test, at least *p* <  0.05 was considered significantly different when compared with saline group.

**Table 2:** Systemic cytokine/chemokine levels after four hours i.p. IL-1β injection in the offspring at four-week old age.

mean±S.E.M (pg/mL) Treatment	IL-6	KC	MCP-1	IL-1α	IL-1β
Saline	3.85 ± 2.56	59.37 ± 18.75	13.26 ± 7.34	116.8 ± 15.01	0.5 ± 0.02
IL-1β (i.p.)	364.5 ± 252.1	9355 ± 3796	2877 ± 719.4*	129.9 ± 11.86	5.55 ± 2.69

Results are presented as: mean ± S.E.M, n = 3. *comparison was performed with t-test, at least *p*  <  0.05 was considered significantly different when compared with saline group.

**Figure 1:**
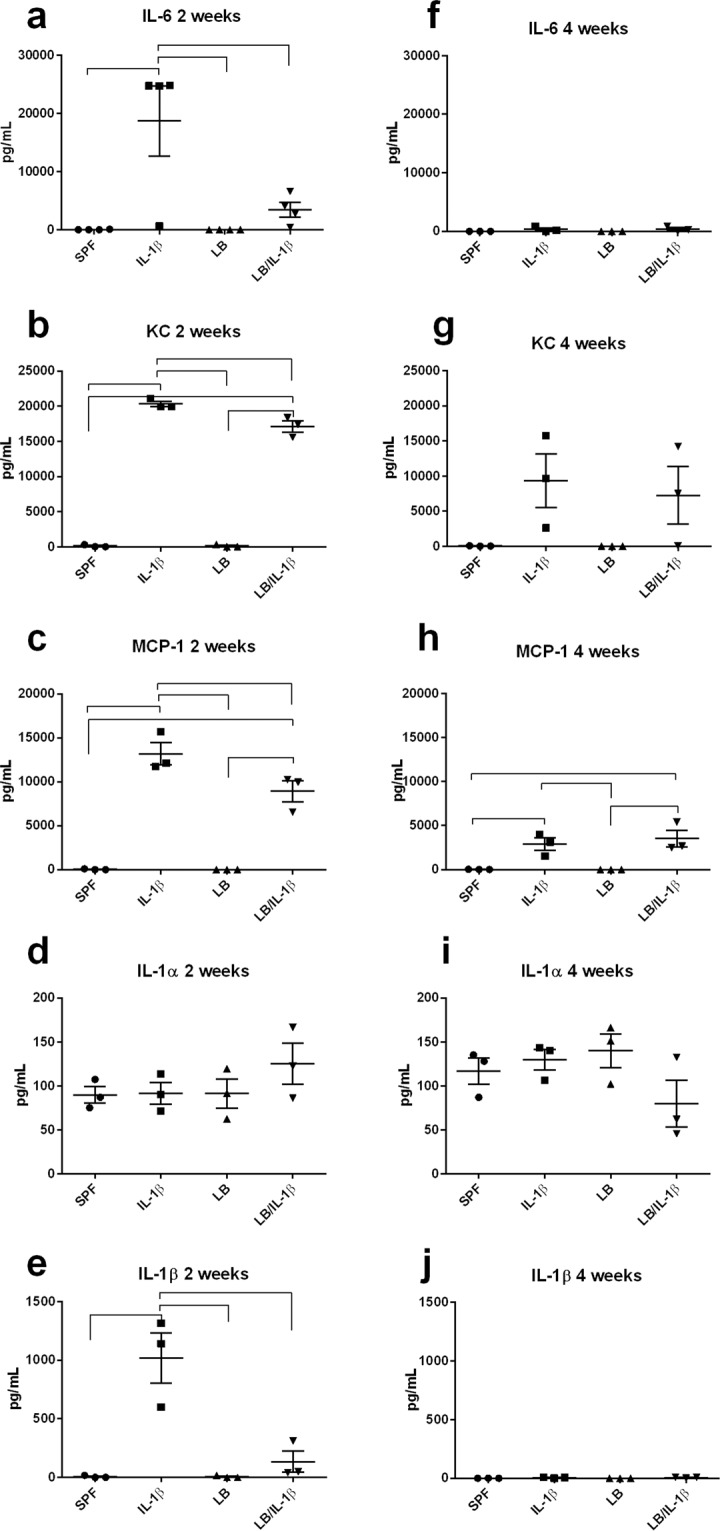
Maternal LB treatment significantly reduces systemic inflammation induced by postnatal IL-1β insult in the offspring. Systemic levels of IL-6 (**a**), KC (**b**), MCP-1 (**c**), IL-1α (**d**), and IL-1β (**e**) at two weeks of age (at least n = 3) and IL-6 (**f**), KC (**g**), MCP-1 (**h**), IL-1α (**i**), and IL-1β (**j**) at four weeks of age (n = 3) were measured by ELISA in serum four hours after saline or IL-1β (i.p.) injection. Data were presented as mean ± SEM. Bars with ⎴ denote significant difference between experimental groups (at least *p* < 0.05).

We then investigated whether maternal supplementation of LB reduces systemic responses to IL-1β insult in the offspring. The baseline circulating levels of IL-6, KC, MCP-1, IL-1α, and IL-1β were not statistically significantly different in the maternally LB supplemented vs unsupplemented offspring at either two or four weeks of age (Fig. 1a–j, *p* > 0.05, n = 3). However, maternal treatment with LB significantly downregulated the IL-1β-induced systemic levels of IL-6, KC, MCP-1, and IL-1β (Figs. 1a–c,e) at two weeks of age, by one-way ANOVA with Tukey’s post *hoc* analysis (at least *p* < 0.05, n = 3). Maternal LB supplementation did not affect the IL-1β-induced increased MCP-1 levels in the offspring at four weeks of age (Fig. 1h, *p* > 0.05, n = 3).

### Maternal LB exposure rescued IL-1β -induced blood-brain barrier (BBB) dysfunction in two weeks old offspring

**Figure 2:**
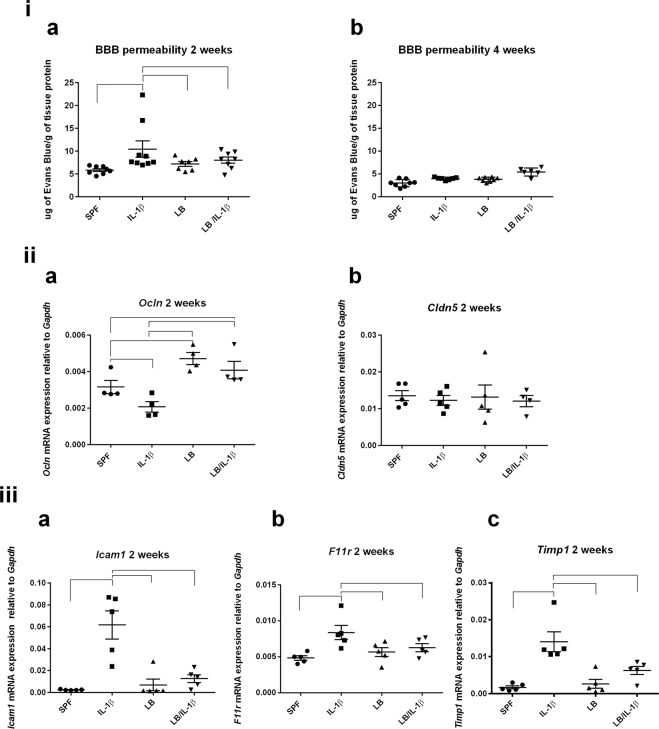
Maternal LB supplementation regulates several BBB characteristics in the offspring. (i). Intraperitoneally administered Evans blue dye (4 ml/kg, 2% [w/v]) in saline appeared significantly higher in the homogenized brains following the injection of IL-1β compared to the control group administered with saline only at two weeks of age (n = 8–9, *p* < 0.05) (**a**), but not at four weeks of age (n = 7–8) (**b**). Increased BBB permeability in two weeks old IL-1β-treated offspring was prevented by maternal LB treatment (n = 7–8, *p* < 0.05). (ii) Maternal LB supplementation promoted *Occludin* gene expression and prevented IL-1β-induced decreased *Occludin* expression in the two-week old offspring. Transcripts of Occludin (*Ocln*) (n = 4, (a) and Claudin-5 (*Cldn5*) (n = 4–5, (b) were evaluated by RT-PCR in the cerebral cortex of the two weeks old offspring in both the maternally un-supplemented and supplemented groups after four hours of saline or postnatal IL-1β injection. (iii) Maternal exposure of LB blocked IL-1β-induced expression of markers for vascular injury, leukocyte recruitment, and ECM integrity. Transcripts of *Icam1* **(**n = 5, (a), *F11r* (n = 5, (b) and *Timp1* (n = 5, (c) were evaluated by RT-PCR in the cerebral cortex of the two weeks old offspring in both the maternally un-supplemented and supplemented groups after four hours of saline or postnatal IL-1β injection. For (ii) and (iii), data were normalized to *Gapdh* gene expression and presented as mean ± SEM. For (i), (ii), and (iii), Bars with ⎴ denote significant difference between experimental groups (at least *p* < 0.05).

Studies have suggested that systemic inflammation can disrupt BBB integrity^58^. We evaluated BBB permeability of cerebral blood vessels, measured by extravasation of Evans blue dye. A two-way ANOVA analysis was first conducted to examine the effects of age (two weeks vs four weeks) and proinflammatory insult (without vs with) on BBB permeability in SPF mice (see Supplemental Fig. S1). There was not a statistically significant interaction between the effects of age and proinflammatory insult on BBB permeability, *F* (1, 28) = 2.891, *p* = 0.1002. Sidak’s multiple comparisons test revealed that IL-1β (i. p.) induced a significant increase in Evans blue leakage into the mouse brain of two (pre-wean), but not four (post-wean), weeks old mice (Fig. S1, *p* < 0.01, for 2 weeks n = 8–9 and *p* > 0.05, for four weeks n = 7–8). This result is consistent with previous reports that early postnatal rodents are more susceptible to systemic inflammation-induced BBB permeability (pre-wean in rodents, a stage of brain development equivalent to 22–42 weeks of gestation in humans^59,60^). Maternal exposure to LB normalized the IL-1β-induced increased BBB permeability at two weeks of age (Fig. 2i a, n = 7–8). Neither maternal supplementation with LB nor IL-1β (i. p.) had impact on BBB permeability at four weeks of age (Fig. 2i b, n = 6–8, *p* > 0.05).

Since we observed most of the systemic inflammatory responses and increased BBB permeability in the two week old mice, the remainder of our investigations focused on this more vulnerable pre-wean population. The IL-1β-induced increased permeability in two-week old mice was associated with reduced cerebral gene expression of the tight junction (TJ) protein *Occludin (Ocln)* (Fig. 2ii a, *p* < 0.001, n = 4) but not *Claudin‐5* (*Cldn5*, Fig. 2ii b, *p* > 0.05, n = 4–5), evaluated by RT-PCR. LB maternal supplementation significantly increased the expression of *Ocln* without postnatal inflammatory insult (Fig. 2ii a, *p* < 0.0001, n = 4) and the *Ocln* levels in the offspring remained significantly higher than unsupplemented pups even after IL-1β treatment (Fig. 2ii a, *p* < 0.001, n = 4).

### Maternal LB exposure regulated IL-1β-induced leukocyte recruitment and extracellular matrix (ECM) damage in two-week old offspring

Leukocyte recruitment and extracellular matrix (ECM) damage are two other signature processes of compromised BBB integrity. Both intercellular adhesion molecule-1 (*Icam-1*) and junctional adhesion molecule-1 (*F11r*) serve as ligands for lymphocyte function-associated antigen-1 (LFA-1) to regulate leukocyte transendothelial migration known as diapedesis into the CNS^61,62^. The tissue inhibitors of metalloproteinases (TIMPs) are tissue specific, endogenous inhibitors of metalloproteinases, including the matrix metalloproteinases (MMPs), and regulate ECM proteolysis and turnover^63^. IL-1β (i.p.) increased the expression of brain *Icam-1* (Fig. 2iii a, *p* < 0.0001, n = 5), *Jam-1*/*F11r* (Fig. 2iii b, *p* < 0.0001, n = 5), and *Timp1* expression (Fig. 2iii c, *p* < 0.0001, n = 5), hallmarks for vascular injury, leukocyte recruitment and ECM injury. Maternal exposure to LB completely blocked the IL-1β-induced increased *Icam-*1 and *F11r* expression (Fig. 2iii a and 2iii b, *p* < 0.0001, n = 5; respectively). Offspring of the maternal supplemented group challenged by IL-1β had significantly lower *Timp1* gene expression when compared to the offspring challenged by IL-1β without maternal exposure to probiotics (Fig. 2iii c, *p* < 0.05, n = 5). Taken together, these data demonstrate that maternal LB exposure protected against systemic inflammation-induced BBB dysfunction in the offspring by regulating tight junction disruption, vascular injury, leukocyte recruitment and ECM repair.

### Maternal administration of LB attenuated postnatal IL-1β-induced astrocyte and microglia activation

**Figure 3:**
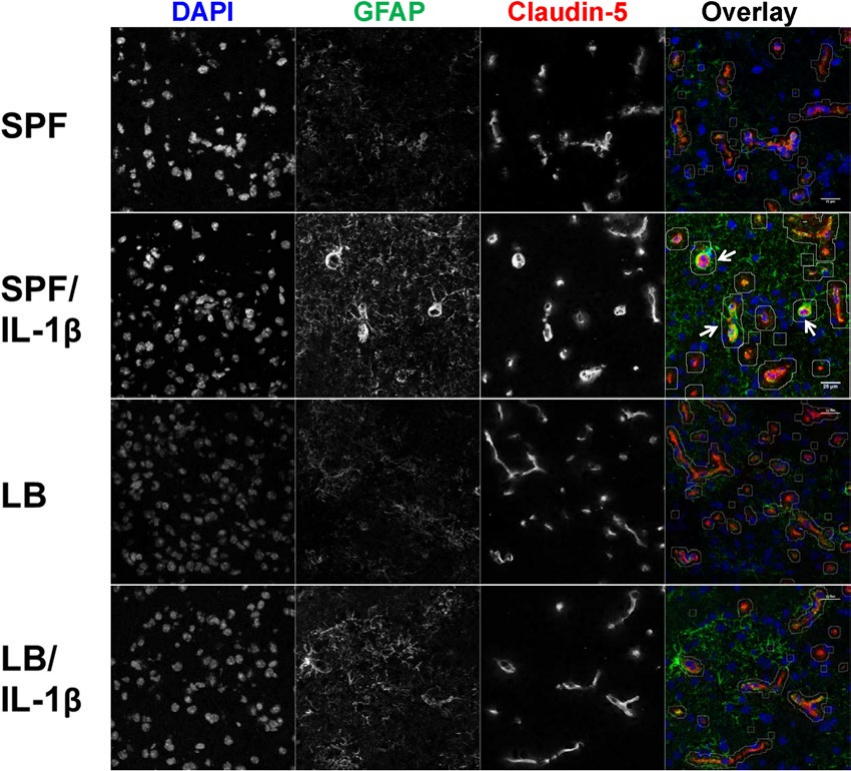
Increased astrocyte activation in IL-1β-stimulated two-week old offspring is reduced by maternal LB supplementation. Representative images of fluorescence microscopy of claudin-5^+^ (location of the brain capillaries, red), GFAP^+^ astrocyte (green), and DAPI (nuclei, blue). Three to five sections per mouse were examined and at least three mice were examined in each group. Stronger than control SPF GFAP staining was observed around the blood vessel (see arrow) after IL-1β insult. Maternal supplemented group (LB) with or without postnatal insult had GFAP levels similar to the control group indicating that LB supplementation prevented astrocyte activation around the BBB endothelium.

**Figure 4:**
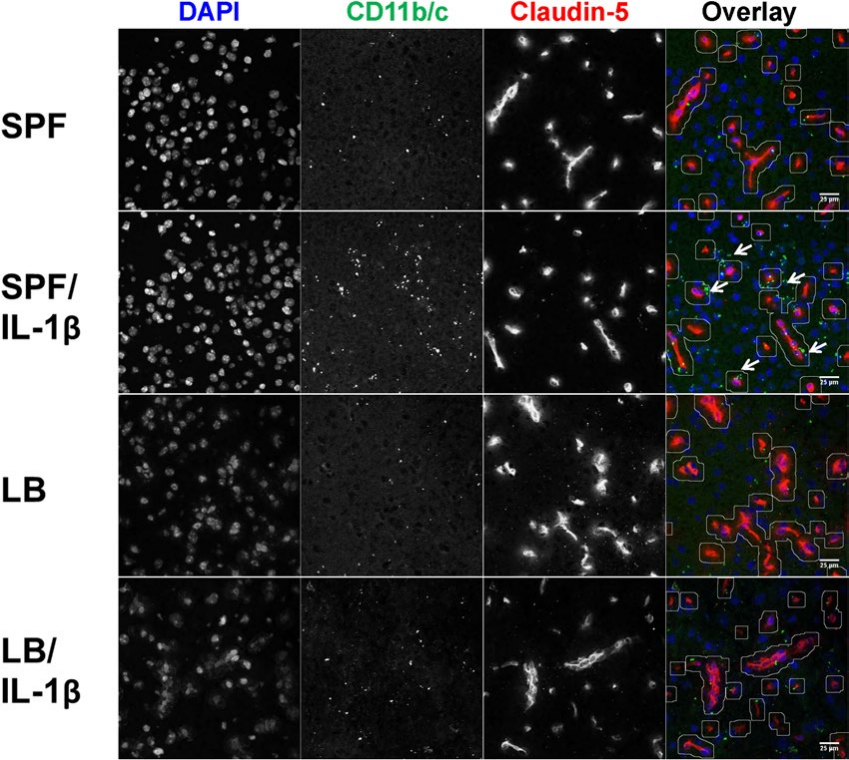
Increased microglia activation in IL-1β-stimulated two-week old offspring is reduced by maternal LB supplementation. Representative images of fluorescence microscopy of claudin-5^+^ (location of the brain capillaries, red), CD11b/c^+^ microglia (green), and DAPI (nuclei, blue). Three to four sections per mouse were examined and at least four mice were examined in each group. The CD11b/c^+^ staining of the maternally probiotic supplemental group remained similar to the un-supplemented group in term of both pattern (scattered) and intensity (see Fig. 5). However, IL-1β stimulated the recruitment of microglia to the BBB, demonstrated by increased CD11b/c^+^ staining around the BBB endothelium (see arrow). Maternal LB supplementation reduced the IL-1β-induced microglia translocation to the vicinity of BBB.

**Figure 5:**
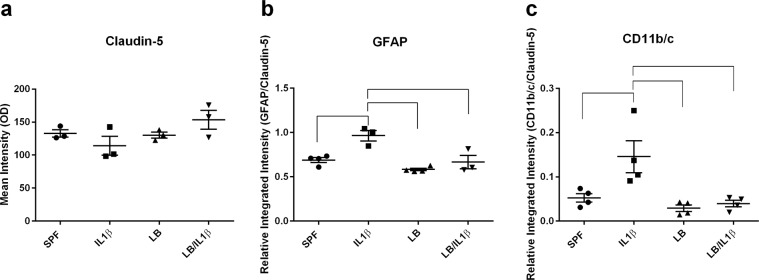
Quantification of astrocyte and microglia activation using ImageJ (NIH). Overall expression of claudin-5 was not affected by LB supplementation or inflammatory insult (**a**). Thus, the GFAP and CD11b/c expression in the vicinity of the blood vessel is expressed as GFAP (n = 3–4, (**b**) or CD11b/c (n = 4, (**c**) levels over claudin-5 levels in the selected blood vessel area. Bars with ⎴ denote significant difference between experimental groups (at least *p* < 0.05).

To determine whether changes in circulating cytokine levels and BBB integrity induce astrocyte activation and microglial recruitment to cerebral vasculature, we performed immunohistochemical staining for glial fibrillary acidic protein (GFAP)^+^ astrocytes (Fig. 3, green) and CD11b/c^+^ microglia (Fig. 4, green). Brain blood capillaries were defined by co-staining with a BBB-specific TJ protein claudin-5 (Figs. 3 and 4, red). Consistent with the gene expression data (Fig. 2ii b), we did not find any differences in claudin-5 (red, Figs. 3 and 4) protein levels in the cerebral cortex of the offspring from either the LB-supplemented or un-supplemented group with or without postnatal peripheral IL-1β insult. Integrated intensity levels of claudin-5 were obtained from analysis using ImageJ (NIH) and are presented in Fig. 5a (*p* > 0.05, at least n = 4). Systemic inflammation induced by IL-1β (i. p.) resulted in significantly increased GFAP staining (green) around the blood vessel (identified by claudin-5 staining, red), indicating a strong astrocyte activation in the BBB (Fig. 3), with quantification presented as GFAP intensity over claudin-5 measurement in Fig. 5b (*p* < 0.01, n = 4). Maternal LB exposure did not change the GFAP expression (Fig. 3) but attenuated IL-1β-induced increased astrocyte activation (Fig. 3). Systemic inflammation caused by IL-1β (i. p.) also induced microglia recruitment and activation around the blood capillaries (Fig. 4). Maternal LB exposure attenuated IL-1β-induced increased CD11b/c expression around the blood vessels, indicating reduced microglia recruitment to the vasculature (Fig. 4, *p* < 0.05, n = 4). Quantitative analysis CD11b/c is presented in Fig. 5c using ImageJ and expressed as integrated intensity levels of CD11b/c over claudin-5.

### Maternal administration of LB ameliorated postnatal IL-1β-induced neuroinflammation

**Figure 6:**
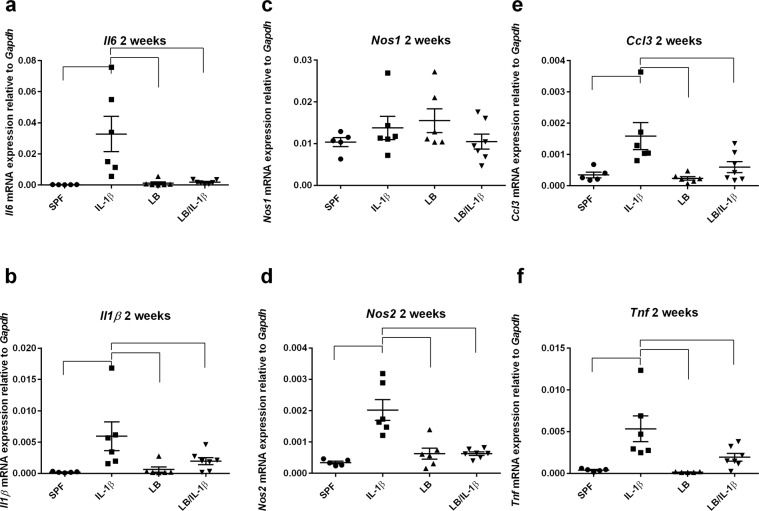
Systemic inflammation-induced neuroinflammation is blocked in the two-week old offspring of maternal LB supplemented mice. Neuroinflammation markers *Il6* (**a**), *Il1β* (**b)**, *Nos2* (**d**), *Ccl3* (**e**) and *Tnf* (**f**), but not *Nos1* (**c**), evaluated by RT-PCR, were upregulated by i.p. injection of IL-1β after four hours in two weeks old mice (n = 5–6). Maternal exposure of LB blocked the Il-1β induced increased expression of these markers (n = 6–7). Data were normalized to *Gapdh* gene expression and are presented as mean ± SEM. Bars with ⎴ denote significant difference between experimental groups (at least *p* < 0.05).

To further investigate the effects of maternal exposure to LB on offspring brain inflammatory status, we evaluated biomarkers of neuroinflammation in the cerebral cortex tissues at two weeks of age by RT-PCR. As depicted in Fig. 6, maternal LB did not change the steady-state inflammation status of the offspring brain. IL-1β injection (i. p.), when compared to saline, resulted in robust increases in cerebral cortex mRNA expression of *Il6* (Fig. 6a), *Il1β* (Fig. 6b), *Nos2* (Fig. 6d), *Ccl3* (Fig. 6e), and *Tnf* (Fig. 6f), but not *Nos1* (Fig. 6c) in the offspring at two weeks of age (at least *p* < 0.01, n = 5-6). Maternal exposure to LB completely mitigated the Il-1*β*-induced *Il6, Il1β*, *Nos2*, *Ccl3*, and *Tnf* gene expression increases (at least *p* < 0.05, n = 6-7; Tukey’s post *hoc* test after one-way ANOVA).

### Maternal administration of LB regulated neuroinflammation through modulation of transcriptional factors

**Figure 7:**
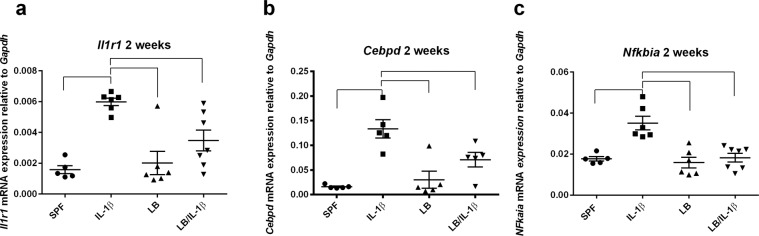
Regulation of neuroinflammation by maternal LB supplementation in the two-week old offspring is mediated by transcriptional regulators. Transcripts of the IL-1β receptor *Il1r1* (**a**) and the transcriptional regulators *Cebpd* (**b**) and *Nfkbia* (**c**) were evaluated by RT-PCR. *Il1r1* gene expression was not affected by maternal LB supplementation (n = 5–6, (**a**). *Cebpd* (**b**) and *Nfkbia* (**c**) were upregulated by i.p. injection of IL-1β after four hours (n = 5-6). Maternal exposure of LB blocked the Il-1β-induced increased expression of these markers (n = 6–7). Data were normalized to *Gapdh* gene expression and are presented as mean ± SEM. Bars with ⎴ denote significant difference between experimental groups (at least *p* < 0.05).

To delineate the potential mechanisms by which maternal LB supplementation modulates IL-1β-induced neuroinflammation in the offspring, we first investigated whether the reduced responses in the offspring brain were due to the downregulation of interleukin-1 receptor (IL1r). As demonstrated in Fig. 7a, there was no difference in *Il1r1* gene expression between the control and the maternal supplemented groups. We then determined the transcriptional regulators in the brain responsible for the observed proinflammatory profile. Both CCAAT/enhancer-binding protein delta (CEBPD)^64–71^ and NF-κB/IκB^72,73^ pathways have been shown to be master transcriptional regulators of inflammatory responses. We found that postnatal insult with IL-1β (i. p.) significantly increased the mRNA expression of *Cebpd* and *Nfkbia* (which encodes IκBα) in the cortex of the mice at two weeks old of age (Fig. 7b,c, respectively, at least *p* < 0.01, at least n = 4). LB maternal supplementation did not change the baseline transcriptional regulators expression when compared to the unsupplemented controls and significantly ameliorated the IL-1β-induced *Cebpd* and *Nfkbia* gene expression levels (Fig. 7b,c, respectively, at least *p* < 0.01, at least n = 4). Additionally, Pearson correlation analysis revealed that there were strong statistically significant correlations between *Cebpd* (Fig. S2A), or *Nfkbia* (Fig. S2B), and neuroinflammation gene expression levels (see *r* values and *p* values under each plot), with the exception of two comparisons: *Nfkbia* or *Cebpd* versus brain levels of *Nos1*. Together, these data suggest that modulation of IL-1β-induced neuroinflammation in the offspring by maternal LB exposure could be due to regulation of the transcriptional regulators CEBPD and IκBα.

### Maternal administration of LB promoted the development of neurons and oligodendrocyte precursor cell (OPC) in the offspring

**Figure 8:**
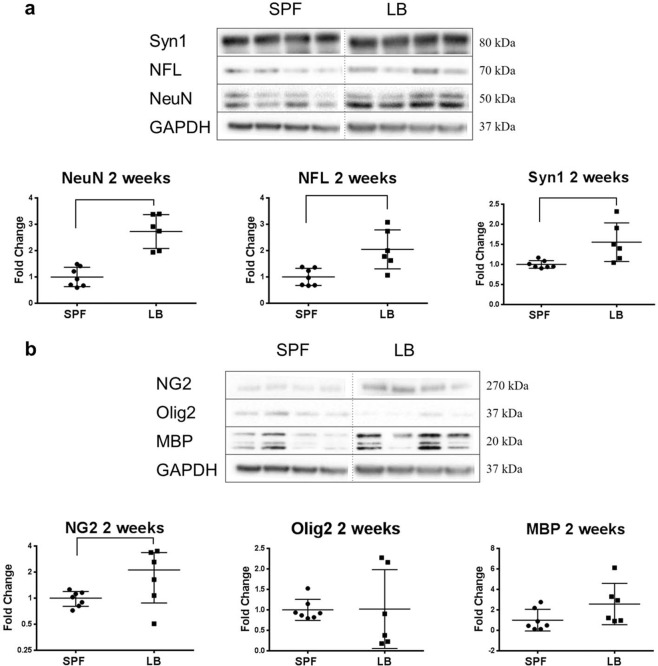
Maternal LB exposure promotes neuronal and oligodendrocyte precursor cell development in the offspring. Proteins of two weeks old cortex tissues were separated on SDS-PAGE gels and probed with NeuN, NFL and Syn1 for neuronal development (**a**) and NG2, Olig2, MBP for oligodendrocyte development (**b**). For both (**a**) and (**b**), top: representative raw images of western blot analysis; bottom: scatter plots of densitometry results normalized to GAPDH displayed as a fold of control. Unpaired t test with Welch’s correction was used to detect the difference between SPF (n = 7) and LB (n-6) group. NeuN-Neuronal Nuclei; NFL-Neurofilament-L; Syn1-Synapsin; NG2-Neuroglia antigen 2; Olig2-Oligodendrocyte Transcription Factor 2; MBP-Myelin Basic Protein. Bars with ⎴ denote significant difference between experimental groups (at least *p* < 0.05). Blots were cropped and the original blots are presented in Supplementary Figure S3.

We have previously shown that a microbial community from a poor growth preterm infant was associated with delayed neuronal and oligodendrocyte development and myelination in the early (two weeks of age) postnatal brain^20^, thus we next investigated the effects of maternal LB supplementation on the expression levels of NeuN, a postmitotic mature neuron marker; neurofilament protein-L (NFL), marker for neuronal axon growth; Synapsin 1 (Syn1), marker for synapse formation; neuroglia antigen 2 (NG2), marker for OPCs; oligodendrocyte transcription factor (Olig2), marker for pre-myelinated oligodendrocytes; and myelin basic protein (MBP), marker for oligodendrocyte and white matter maturation; respectively. As demonstrated in Fig. 8a with representative western blots on the top panel and quantification on the bottom panel, LB maternal exposure significantly increased the expression of NeuN, NFL, and Syn1 (2.72, 2.05, 1.55 times of the control SPF; respectively) when compared to the control group (*p* < 0.05, n = 7 in SPF, n = 6 in LB group). LB maternal supplementation did not affect the expression of Olig2 and MBP; however, NG2 expression in the maternal LB group was found to be 2.12 times of the control group (Fig. 8b, *p* < 0.05, n = 7 in SPF, n = 6 in LB group). These data demonstrate that maternal LB promotes neuronal and oligodendrocyte precursor cell development in the brains of the offspring.

## Discussion

The human microbiome has increasingly been shown to be an important regulator of CNS development and functions^19,31,56,74^. The effect of the initial establishment of the microbiome in neonates has been associated with basic processes such as neurogenesis, myelination, the establishment of the blood-brain barrier, and microglia maturation as well as with behavioral responses^18,20,74–76^. Preterm delivery disrupts important *in utero* brain maturation^31,56^. Dysbiosis, or altered gut bacterial composition, predisposes preterm infants to inflammatory diseases and infections such as NEC and sepsis that have long term adverse neurological outcomes^14^. Therefore, studies have explored means to modify the preterm microbiome to improve neonatal outcomes. With direct supplementation of probiotics to preterm infants, studies have demonstrated reduced NEC incidence, decreased length of the hospital stay, increased tolerance to feeding, and reduced mortality rate in the preterm population^34^. However, the Food and Drug Administration has not approved the usage of probiotics for the prevention and treatment of disease due to the lack of regulation of strains, dose, duration and quality of probiotics and reported cases of sepsis^34,40,41^. Maternal probiotic supplementation has been shown to be safe and effective to reduce NEC, atopic diseases and IgE-associated allergy in the offspring^47–49,56^, therefore in this study we investigated whether maternal probiotic supplementation can have neuroprotective effects from postnatal inflammatory insult or can have direct impacts on the brain development in the offspring. We first demonstrated in this study that maternal probiotic (LB) supplementation can change systemic immune responses in the offspring with suppression of circulating pro-inflammatory cytokines/chemokines, including IL-6, KC, MCP-1, and IL-1β, after postnatal peripheral inflammatory insult. This protection was then found not only in the blood but also extended to the brain. Postnatal peripheral inflammatory stimuli-induced BBB dysfunction, astrocyte and microglial activation, and neuroinflammation were normalized by maternal LB supplementation. Furthermore, we demonstrated that the regulation of neuroinflammation in the offspring by maternal LB supplementation was mediated through transcriptional regulators such as CEBPD and IκBα. Lastly, maternal LB supplementation promoted neuronal development evidenced by increased expression of NeuN, NFL, and Syn1 and oligodendrocyte progenitor cell development evidenced by increased NG2 expression in the offspring brain. These findings support the current paradigm to beneficially shift systemic immunity through probiotics administration, but this study is the first to demonstrate neuroprotective beneficial effects and the promotion of brain development in the offspring through maternal supplementation from pregnancy to weaning instead of direct administration to the pups.

Elevated circulating inflammatory cytokine levels are associated with a variety of medical conditions in infants. For example, postnatal episodes of inflammation with elevated proinflammatory IL-6 and TNF levels are associated with the development of severe retinopathy of prematurity^77^. Proinflammatory circulating cytokines IL-6 and IL-8 are strongly elevated and IL-4, an immune modulator that inhibits the release of proinflammatory cytokines, such as TNF and IL-1β, is suppressed in NEC patients compared to the healthy controls^78^. One of the proposed benefits of probiotics consumption is the ability to modulate the systemic immune function. In our study, maternal LB supplementation from pregnancy to weaning reduced postnatal inflammatory stimuli-induced systemic inflammation by significantly suppressing the circulating proinflammatory cytokines IL-6, KC, MCP-1, and IL-1β. The observed significantly increased circulating cytokines at two weeks of age is consistent with previous studies^24,58^ demonstrating that the acute systemic inflammatory response is associated with a fundamental proinflammatory cytokine profile consisting of IL-6 and IL-1β, and that IL-1β is a potent inducer of systemic inflammation. Our study agrees with the anti-inflammatory characteristics of probiotics but the novelty of our results lies in the fact the immunomodulatory effects of the LB were transferred from mothers to the offspring examined at the pre-weaned stage when the population is more vulnerable to inflammatory insult.

Emerging evidence has demonstrated communication between the immune system and CNS during peripheral inflammation^23,25–28^. Signaling at the BBB appears to be the prominent communication site at which the brain responds to the peripheral inflammation status^27,79,80^. The BBB is the vascular endothelium that tightly governs the interaction between the circulatory system and CNS to allow for proper brain function and provide protection to the CNS from toxins, pathogens, and inflammation^81^. Studies have discovered that systematic inflammation can increase BBB permeability modeled by i.p. injection of IL-1β^24,57,79^ and LPS^82^. There seems to be a specific window of susceptibility to systemic insults to the BBB since systemic inflammation-induced increased permeability of cerebral blood vessels was only observed in rats before postnatal age of 20 (P20, pre-wean)^57^, but not at P28 (post-wean). Our results are in agreement with these studies demonstrating that IL-1β (i.p.) induced increased BBB permeability at two weeks of age (P14), but not four weeks of age (P28), indicating a developmental maturation of BBB from pre-weaned to post-weaned age in mice.

Studies have further revealed that microbial communities can modulate BBB integrity^75,83^. Compared to SPF mice with normal gut flora, GF mice display increased BBB permeability and reduced expression of the TJ proteins occludin and claudin-5. Exposure of GF mice to a commensal microbiota or monocolonization with C. *tyrobutyricum* or B. *thetaiotaomicron* decreases BBB permeability and normalizes the expression of TJ proteins^75^. In this study, we have shown that maternal LB modulates BBB function in the offspring brain evidenced by normalized increased BBB permeability and decreased *Ocln* gene expression in response to postnatal inflammatory insult-induced systemic inflammation. Maternal LB supplementation resulted in a significant increase in *Ocln* gene expression compared to the unsupplemented group, suggesting that the protective effect of maternal LB supplementation on BBB is not only due to the suppression of systemic inflammation but also to upregulation of the TJ protein occludin itself.

In the presence of systemic inflammation signals, activation of the cerebral endothelium in the BBB by cytokines results in increased endothelial cell expression of adhesion factors such as ICAM1 and redistribution of JAM-1 (F11r) from the TJs^62,84^. The activated ICAM1 and JAM-1(F11r) both serve as ligands for LFA-1 and lead to adhesion, arrest and ultimately extravasation of leukocytes through the endothelium and into the brain^84^. In particular, the efficiency of this extravasation mediated by LFA-1 and ICAM-1 remained low unless the endothelium was stimulated by various inflammatory stimuli in mouse models of experimental autoimmune encephalomyelitis^85^ and liver disease^25,86^. In our study, we discovered that i.p. injection of IL-1β induced the expression of *Icam1* and *Jam-1* (*F11r*) expression in the offspring and maternal supplementation of LB significantly inhibited postnatal IL-1β-induced *Icam1* and *Jam-1* (*F11r*) expression. Furthermore, among the four tissue inhibitors of metalloproteases, TIMP-1 is the inducible form and is up-regulated by proinflammatory factors such as IL-1β and IL-6^87^. It has been hypothesized that acute immune activation of astrocytes increases TIMP-1 in an attempt to repair and protect whereas chronic inflammation down-regulates TIMP-1 impairing the ability of TIMP-1 to preserve ECM^88^. In our study, an acute inflammatory response was modeled by i.p. injection of IL-1β and termination of the experiment four hours after injection. We observed that *Timp1* gene expression was induced by postnatal IL-1β insult but that maternal LB supplementation maintained *Timp1* at a steady-state level and prevented IL-1β-induced *Timp1* increase in the acute inflammation state. Together, these data demonstrate that maternal LB supplementation can mediate the signaling of leukocyte extravasation and ECM homeostasis in the offspring in response to postnatal inflammatory insult.

Strong evidence suggests that peripheral inflammation can result in an inflammatory response within the brain, characterized by synthesis and action of cytokines in the CNS^89–94^. A single dose of a peripheral proinflammatory insult activated microglia and increased prolonged expression of MCP-1, IL-1β, and NF-κB in an adult model of neurodegenerative disease^91^. Additional astrocyte loss and structural changes^95^ and astrocyte gene transcription with a proinflammatory profile in response to peripheral inflammation were also reported^96^. The host microbiota’s role in shaping the brain’s innate immune system was recently reported by a study^76^ showing that GF mice displayed global defects in microglia with altered cell proportions and an immature phenotype, leading to impaired innate immune responses. Previous studies have further demonstrated that probiotics such as VSL#3 reduced systemic immune activation and cerebral monocyte infiltration and attenuated sickness behaviors associated with peripheral liver inflammation^55^. In our study, GFAP and CD11b/c immunostaining demonstrated that maternal LB exposure attenuates postnatal peripheral IL-1β-induced recruitment to the site adjacent to the BBB in the brain and activation of astrocytes and microglial cells. These results provide evidence that there is a signaling pathway between LB-mediated peripheral inflammation, BBB and the brain immune system.

We further demonstrated that postnatal IL-1β insult caused severe neuroinflammation marked by robustly increased *Il6*, *Il1β*, *Nos2*, *Ccl3*, and *Tnf* expression. Endothelial cells at the BBB, the recruited inflammatory cells from the blood, as well as activated microglia, pericytes, and astrocytes are all potential sources of IL-1β, IL-6, MIP-1α (CCL3), and TNF contributing to neuroinflammation^94,97–103^. Furthermore, non-immune cell-produced cytokines can in turn trigger further microglial activation and neuroinflammation^104^. Maternal LB supplementation significantly ameliorated the induced expression of neuroinflammation markers in our study. Other strains of probiotics have been shown to have an anti-inflammatory effect in the brain. Supplementation of *L. helveticus* downregulated inflammatory markers such as NOS2, prostaglandin E2, and IL-1β in the brain^105^. A multi-strain probiotics treatment consisting of *L. helveticus* R0052, *L. plantarum* R1012, and *B. longum* R0175 improved chronic mild stress (CMS)-induced anxiety- and depressive-like behaviors in a mouse model of CMS and ameliorated CMS-induced TNF increase in the brain^106^. Supplementation of LB has been widely used to reduce inflammatory bowel diseases especially in preterm infants with NEC^35,36^. Together with the systemic inflammation results, our study demonstrates that LB is anti-inflammatory and that these immunomodulation properties of LB: 1) can be transferred from the mother to the offspring 2) can be extended from the systemic system to the CNS of the offspring.

Since the observed peripheral inflammation-induced neuroinflammation is at least partially due to the locally produced inflammatory markers evidenced by increased transcriptional levels of these factors, we examined the transcriptional regulators potentially responsible for the up-regulation. As a member of the CCAAT/enhancer-binding protein (C/EBP) family, C/EBP delta (CEBPD) is expressed at relatively low levels under physiological conditions and is upregulated by a variety of extracellular stimuli, such as IL-6^64^, LPS^65^, IL-1β^66,67^, TNF^68^ and interferon-γ^107^. CEBPD can reciprocally induce expressions of cytokines including IL-6, IL-1β, and TNF^69–71,101^. Moreover, enhanced expression of CEBPD plays a critical role in the pathogenesis of inflammatory diseases, such as AD and rheumatoid arthritis^68,108,109^. The expression level of the NF-κB inhibitor NFKBIA (which encodes IκBɑ) is a direct reflection of activation of NF-κB signaling by forming an autoregulatory loop with activated NF-κB transcription factors^72,73^. IκB ɑ protein is degraded upon proinflammatory stimulation, resulting in rapid translocation of NF-κB from the cytoplasm to the nucleus. In turn, IκB ɑ is quickly induced by the activated NF-κB transcriptional pathway^72^. In our study, the peripheral inflammation-induced neuroinflammation was mediated by upregulation of these prototypical proinflammatory transcriptional regulators and maternal LB modulated neuroinflammation in the offspring through downregulation of these factors. Interestingly, IL-1β peripheral insult resulted in the highest increase of *Cebpd* in astrocytes compared to microglia and endothelial cells. These increased astrocytic *Cebpd* expression contributed to the chemoattractant activity and migration and activation of microglia/macrophages in the brain in an animal model of Alzheimer’s disease^101^. Therefore, the observed reduced microglia migration in the offspring by maternal LB supplementation in this study could also be mediated by the *Cebpd* expression.

While mounting studies have demonstrated the impact of the intestinal microbiota on host behaviors such as anxiety and stress in adults and aging related-neurodegeneration^19,23,110^, few studies have investigated the early critical brain development window across prenatal and early postnatal stages. We have previously demonstrated that a microbial community associated with a poor postnatal growth resulted in delayed neuron development using NeuN and NFL expression as markers at the pre-weaned age of P14 and myelination using MBP as a marker at the post-weaned age of P28^20^. In this study, we provide novel observations that maternal supplementation of LB from late gestation to weaning promotes neuron development evidenced by increased NeuN, NFL, and Syn1 expression in the offspring brain. Furthermore, the OPC marker NG2 was also increased by this supplementation. These observations strongly suggest that maternal LB administration can actively modulate brain development in the offspring with therapeutic potential.

We acknowledge that we cannot determine whether the observed neuroinflammation was caused by cytokines crossing the compromised BBB or locally produced cytokines by endothelial cells or brain immune cells such as astrocyte and microglia. As previously discussed, multiple brain cell types including endothelial cells, astrocytes, microglial cells, pericytes, and neurons are all capable producing proinflammatory factors constituting neuroinflammation. We speculate that the deleterious effects in overall neuroinflammation are mediated by a combination of potential pathways: 1) systemic inflammation can directly induce BBB leakage allowing passage of proinflammatory cytokines such IL-1β and IL-6 which then stimulate immune cells and neurons in the brain to produce additional inflammatory cytokines/chemokines. These proinflammatory factors can in turn signal to recruit leukocyte infiltration of the blood vessels to amplify the inflammatory responses; 2) systemic inflammation can induce leukocyte infiltration and/or act directly on endothelial cells of the BBB vasculature. Additionally, a limitation of this study is that we only used CD11b/c to identify macrophages in the brain therefore, it is difficult to distinguish if the activated immune cells were local microglia, which can be identified by CD11b + /CD45^low^ labeling, or infiltrating macrophages, which can be identified by CD11b/c + /CD45^high^ labeling. We provide indirect evidence that leukocyte recruitment might be increased by induced *Icam1* and *F11r* expression, however future studies can elucidate this process further by isolating microglia from the brain and sorting by CD11b/c and CD45 double-labelling using flow cytometry. Finally, investigation of the mechanisms by which maternal LB promotes neuron and OPC development in the offspring is warranted. Future studies will utilize metabolomics analysis to evaluate Sdifferences in the metabolites between the LB-treated group and untreated group to identify potential regulators signaling from the microbiome to the gut to the brain. Epigenetics can also be used to reveal whether maternal LB supplementation genetically programs the offspring to have decreased inflammatory responses and better neurodevelopmental outcomes.

In conclusion, the present study is novel in revealing the impact of the maternal probiotic route on brain development and immune (systemic and neuronal) responses in the offspring. Our data demonstrate that maternal LB supplementation inhibits peripheral inflammation-induced increases in circulating IL-6, KC, MCP-1, and IL-1β levels, BBB dysfunction, cerebral astrocytes and microglial activation, and neuroinflammation and promotes neuron and OPC development in the brain of the offspring. Therefore, targeting the maternal microbiome with probiotics may have a therapeutic role in conferring resilience against pro-inflammatory events and associated impacts on brain health/development.

## Methods

### Ethics approval

All animal procedures were approved by the Institutional Animal Care and Use Committee under the animal protocol No. 71703 and performed strictly in accordance with approved Animal Care and Use Protocols (ACUPs) by the U.S. National Institutes of Health at The University of Chicago.

### Animals

Time pregnant C57/BL6J specific pathogen-free (SPF) mice were obtained from Jackson Laboratory (Bar Harbor, ME, USA) and were kept on a 12-hour light/dark cycle and had access to food and water *ad libitum*. At embryonic days 16 (E16), dams were randomized to be fed daily with either a combination of 10^9^ *Lactobacillus acidophilus* and 10^9^ *Bifidobacterium infantis* (LB) at a ratio of 1:1 from ATCC (No. 53544 and 15697, respectively; Manassas, VA, USA) or vehicle until weaning at postnatal day 21 (P21). Pups delivered naturally and stayed with the mothers until P21. At P14 (two weeks of age) or P28 (four weeks of age), pups were weighed and challenged with intraperitoneal (i.p.) injection of IL-1β (50 ng/g body weight), modeling postnatal systematic inflammation. Groups included both male and female animals. Based on our previous studies^20,74^, sex difference was not prominent in C57BL/6 J mice at this age thus sex effect was not investigated separately. The offspring used in this study were from at least five different litters for SPF mice and six different litters for LB mice. This resulted in four study groups: SPF, IL-1β, LB, and LB/IL-1β. After four hours of IL-1β treatment, mice were sacrificed and serum was collected and stored at −80 °C for cytokine/chemokine multiplex analysis. Tissue harvesting was carried out with left cortex snap frozen for biochemical analysis and right cortex for immunohistochemical analysis.

### Cytokine assay

Multiplex analysis was performed according to the manufacturer’s instructions using a kit for a panel of mouse cytokines/chemokines based on the Luminex xMAP technology with magnetic beads (EMD Millipore Corporation, Billerica, MA, USA). Experiments were performed in triplicate. The kit enables a simultaneous multiplex analysis of 12 cytokines, chemokines, and interleukins in a 25 µl (2x diluted) serum sample.

### Blood-brain barrier (BBB) Permeability Evaluation

Evaluation of BBB permeability was carried out as previously described^111,112^. Briefly, mouse pups receive a 2% solution of Evans Blue (w/v) in normal saline (4 ml/kg of body weight, i.p.), followed four hours later by transcardiac perfusion with 40 ml of ice-cold PBS. Brain tissues were removed from the skull and divided into right and left hemispheres, snap-frozen in liquid nitrogen, and stored at −80 °C. Right hemisphere samples were homogenized in 1000 μl of PBS and centrifuged at 12, 000 × g for 30 mins at 4 °C. 500 μl of the supernatant was mixed with 500 μl 50% trichloroacetic acid, incubated at 4 °C overnight and then centrifuged at 12, 000 × g for 30 mins at 4 °C. Evans Blue stain was measured by ELx800 spectrophotometer (BioTek Instruments, Winooski, VT, USA) at 610 nm and quantified using a standard curve. The results are presented as (μg of Evans Blue stain)/(g of total protein).

### RNA isolation and real-time PCR

Using the RNeasy Plus Mini Kit (QIAGEN GmbH, Hilden, Germany) total RNA from snap frozen brains was isolated. 500 ng of isolated total RNA was used to synthesize cDNA using RT^2^ First Strand Kit from QIAGEN. TaqMan Assays (Thermo Scientific Inc., Waltham, MA, USA) were used for genes of interest and the housekeeping gene *Gapdh*. Gene expression was normalized to the housekeeping gene.

### Immunohistochemistry

Immunostaining protocol was as described previously^20^. Brains were obtained from mice at postnatal age of two weeks and embedded in OCT. Eight μm frozen sections were fixed in ice-cold methanol at −20 °C for 20 minutes. The samples were incubated with blocking solution (5% goat serum) in 0.2% Triton-X PBS (PBST) for one hour at room temperature (RT) after rinsing with 1x PBS three times and permeabilized with PBST for 15 mins. The tissue sections were then incubated with respective 50 μL of primary antibody solution overnight at 4 °C. After wash with PBST four times for 10 mins, the sections were incubated with respective fluorophore-conjugated secondary antibodies for one hour at RT. The sections were counterstained with DAPI-antifade mounting medium (Invitrogen Inc., Carlsbad, CA, USA). Imaging was performed at the University of Chicago Integrated Light Microscopy Facility. Images were captured with a Leica TCS SP8 laser scanning confocal microscope (Leica Microsystems, Inc., Buffalo Grove, IL, USA). Imaging processing and analysis was obtained using ImageJ (U. S. National Institutes of Health, Bethesda, Maryland, USA, http://imagej.nih.gov/ij/, 1997–2012)^113^.

### Western blotting

Immunoblotting protocol was as described previously^20^. Samples were lysed in 300 µL ice-cold 1x RIPA lysis buffer (50 mM Tris-Cl at pH=7.5, 150 mM NaCl, 1% NP-40 alternative (EMD Millipore Corporation), 0.5% (wt/vol) Sodium deoxycholate, 0.1% (wt/vol) SDS with protease inhibitors (Roche Diagnostics GmbH, Mannheim, Germany) and spun at 12,000 g for 10 minutes at 4 °C. Total protein concentration was measured via Pierce BCA Protein Assay (Thermo Scientific Inc.). Equal amounts of protein lysate were subjected to SDS-PAGE electrophoresis using Criterion XT 4–12% Bis-Tris precast gels (Bio-Rad Laboratories, Inc., Hercules, CA, USA) and transferred to PVDF membranes using a semi-dry transfer system (Bio-Rad). The membranes were blocked with 5% nonfat milk (NFM) (Bio-Rad) in Tris-buffered saline (TBS, 0.02 mol/l Tris-HCl, 0.137 mol/l NaCl, pH=7.5) with 0.1% Tween-20 (TBST) for an hour on a shaker at RT. The membranes were cut into pieces depending on the molecular weights of the proteins of interest and then incubated with respective primary antibodies in 5% NFM in TBST overnight at 4 °C. The membranes were washed four times for 10 min with TBST and then incubated with secondary antibodies for one hour at RT. The chemiluminescent signal was developed using SuperSignal West Femto maximum sensitivity substrate (Thermo Scientific) and captured using a Molecular Imager ChemiDoc XRS + imaging system (Bio-Rad). Densities were quantified using Fiji (NIH, Bethesda, MD, USA), normalized to that of GAPDH, and reported as a fold increase to SPF control.

### Antibodies and reagents

Monoclonal Claudin-5 antibody (#35–2500) was purchased from Thermo Fisher Scientific (Rockford, IL, USA). Polyclonal GFAP antibody (#NB300–141) and CD11b/c antibody (#NB 110–70466) were purchased from Novus Biologicals, LLC (Centennial, CO, USA). Neuronal nuclei (NeuN #MAB377), synapsin (Syn1 #AB1543), oligodendrocyte transcription factor 2 (Olig2 #MABN50) and Neural/glial antigen 2 (NG2 #AB5320) antibodies were purchased from EMD Millipore (Billerica, MA, USA). Myelin basic protein (MBP #13344), neurofilament-Light chain (NFL #2837), and GAPDH (#5174) antibody were purchased from Cell Signaling Technology (Danvers, MA, USA). Secondary anti-mouse-HRP and anti-rabbit-HRP antibodies were purchased from Santa Cruz Biotechnology, Inc. (Dallas, TX, USA). Secondary Alex Fluor goat anti-rabbit IgG-488 (H + L) (A11034) and Alex Fluor goat anti-mouse-594 (H + L) (A11032) were purchased from Invitrogen Inc. (Thermo Fisher Scientific). Recombinant murine IL-1β (Cat# 211-11B) was purchased from PeproTech Inc (Rocky Hill, NJ, USA).

### Statistics

Data are presented as mean ± SEM. One-way ANOVA followed by Tukey’s post-*hoc* test for multiple comparison test among all experimental groups was performed when more than two groups were analyzed using GraphPad Prism version 6.07 for Windows, GraphPad Software, La Jolla, California, USA, www.graphpad.com. Unpaired t-test with Welch’s correction for unequal variances was used to detect the difference when only two groups were compared. P value less than 0.05 was considered statistically significant.

## Supplementary information

Supplementary Information.

## Data Availability

The data generated during the current study are available from the corresponding author on reasonable written request.
