# Supplementary material for: Maternal administration of probiotics promotes brain development and protects offspring’s brain from postnatal inflammatory insults in C57/BL6J mice
Source: Sci Rep. 2020 May 18;10:8178. doi: 10.1038/s41598-020-65180-0 (PMC7235088; doi:10.1038/s41598-020-65180-0)

Supplementary Information

Maternal administration of probiotics promotes brain development and protects offspring's brain from postnatal inflammatory insults in C57/BL6J mice

Jing Lu[1], Lei Lu[1], Yueyue Yu[1], Jillian Baranowski[2], Erika C. Claud[1,*]

[1]The University of Chicago, Pritzker School of Medicine, Department of Pediatrics, Chicago, IL, 60637, USA
[2]The University of Chicago, Pritzker School of Medicine, Chicago, IL, 60637, USA

[*] eclaud@peds.bsd.uchicago.edu

Supplemental Figure S1

Figure S1

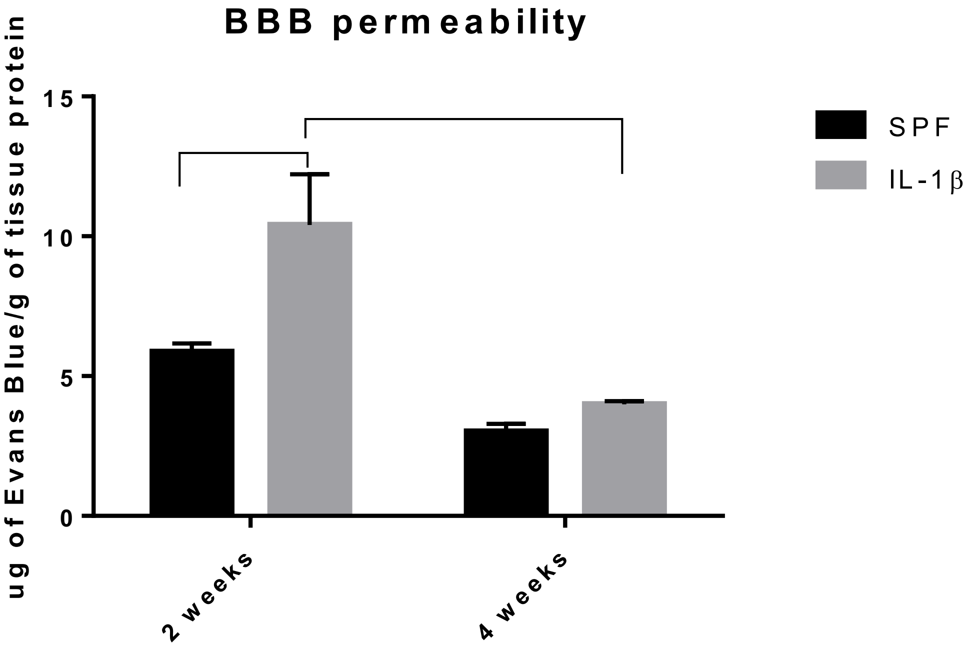

Figure S1. Evans blue permeability assay of two weeks and four weeks old phosphate buffered saline (PBS) or IL-1ß-treated SPF C57BL/6J mice. The intraperitoneally administered EB (4 mL/kg, 2% [w/v] in PBS appeared significantly higher in the homogenized brains following the injection of IL-1ß compared to the control group administered with vehicle (PBS) only at two weeks (*p < 0.05) but not at four weeks of age (two-way ANOVA. Interaction p=0.1002, main effects age p=0.0138 and insult p=0.0001). Data was presented as mean ± SEM.

Figure S2A

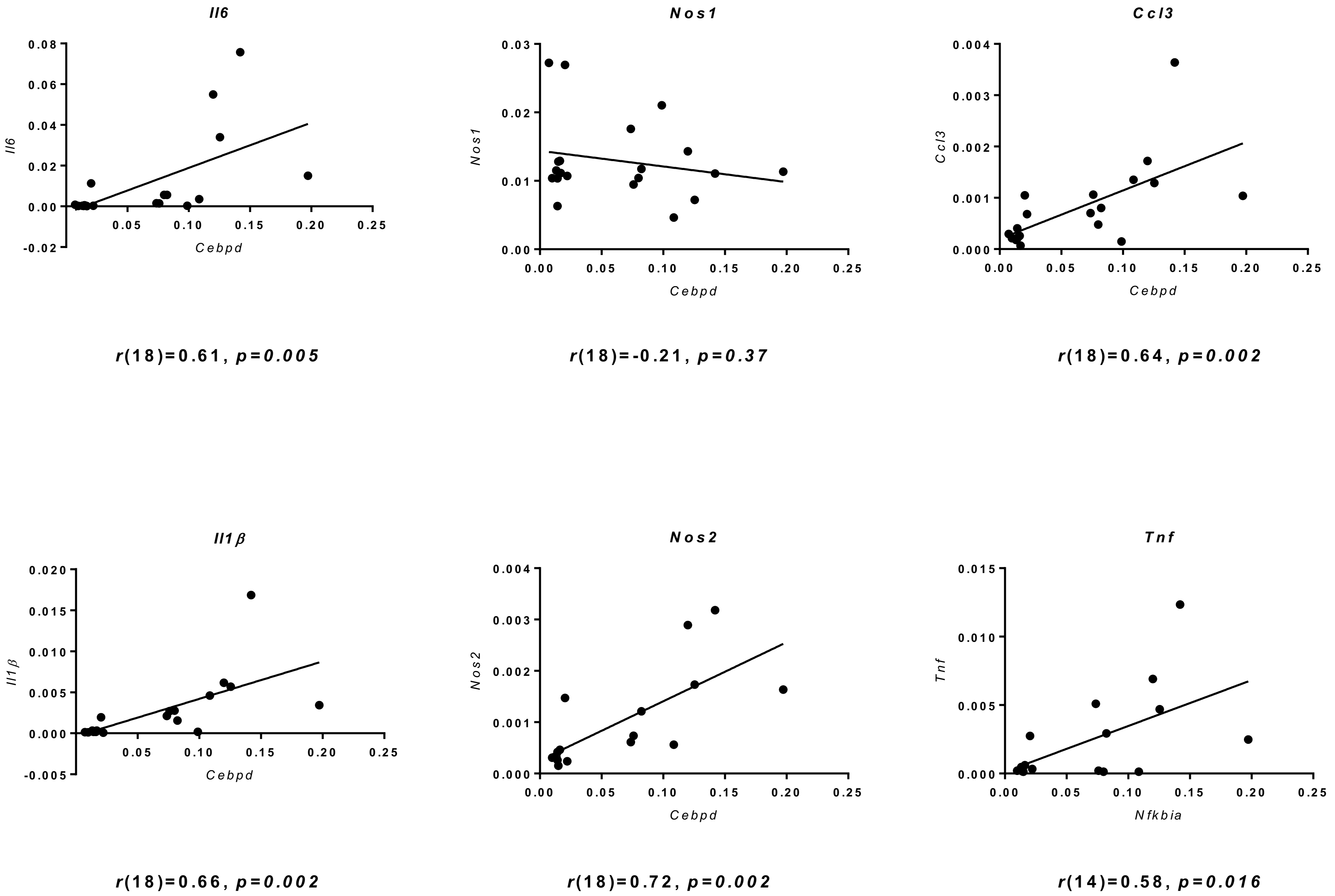

Il6
Il6
Cebpd
r(18)=0.61, p=0.005
Nos1
Nos1
Cebpd
r(18)=-0.21, p=0.37
Ccl3
Ccl3
Cebpd
r(18)=0.64, p=0.002
Il1β
Il1β
Cebpd
r(18)=0.66, p=0.002
Nos2
Nos2
Cebpd
r(18)=0.72, p=0.002
Tnf
Tnf
Nfkbia
r(14)=0.58, p=0.016

Figure S2B

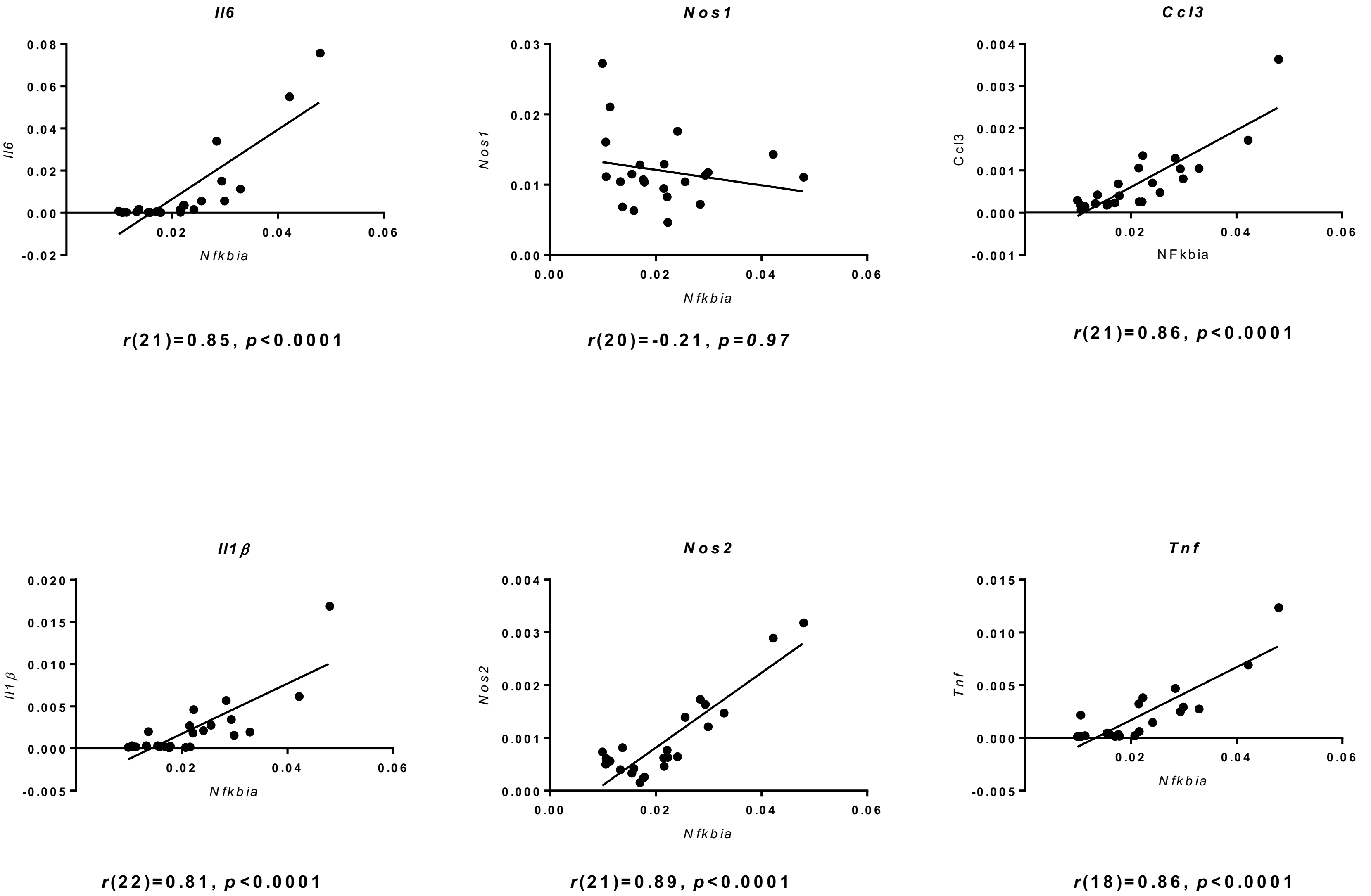

Figure S2. Correlation plots and Pearson's correlation coefficients (r) for *Cebpd* (2A), and *Nfkbia* (2B) versus neuroinflammation markers. There were strong statistically significant correlations for these comparisons (see *r* values and *p* values under each plot), with the exception of two comparisons: *Cebpd* or *Nfkbia* versus brain levels of *Nos1*.

Supplemental Figure S3

Blots with molecular markers illustrated in Figure 8.

Figure 8A

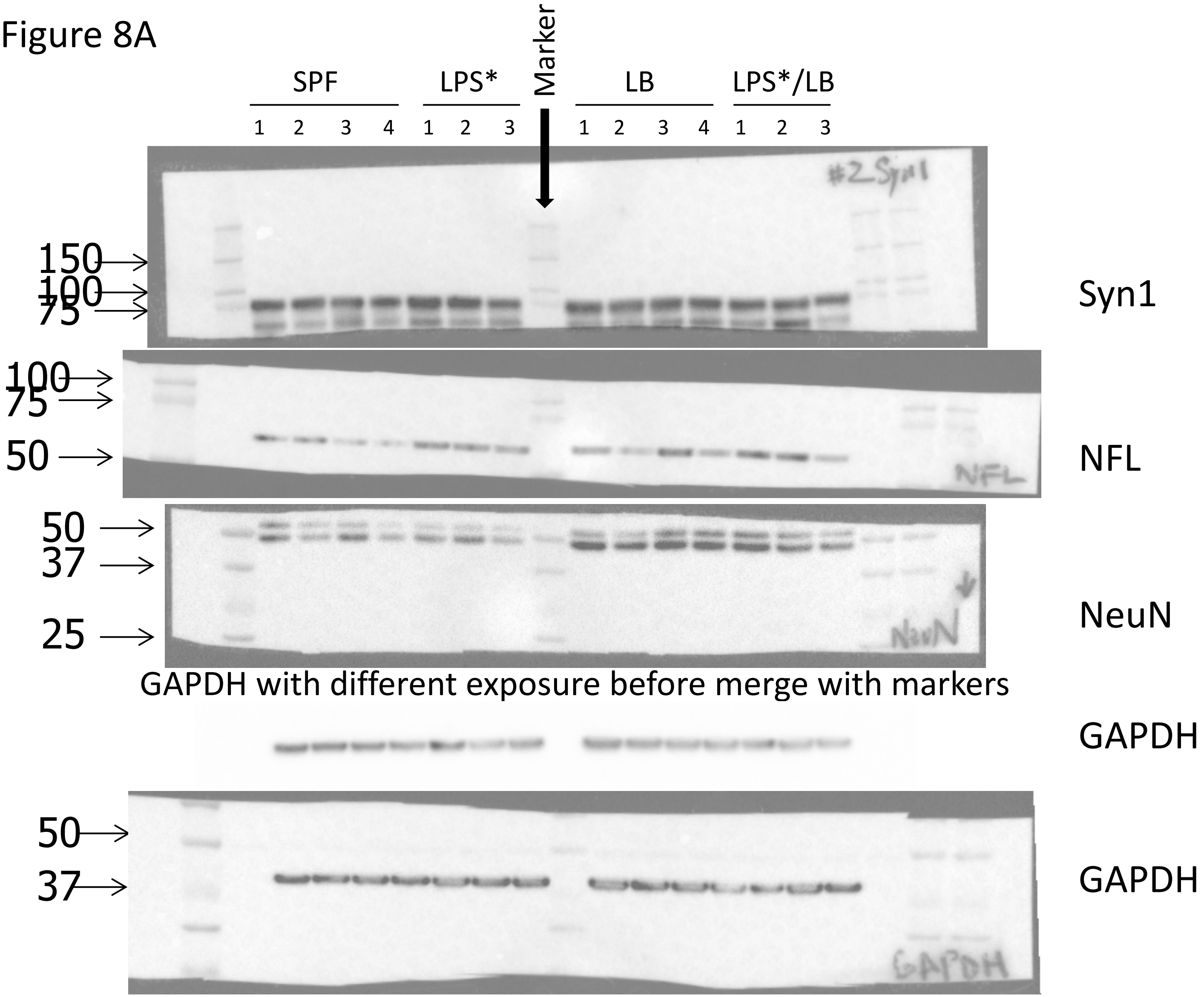

*For this manuscript these blots are used to demonstrate difference between SPF and LB groups. LPS was administrated to the mothers, not to the pups in these sets of blots. Maternal LPS challenge is not the focus of this study.

Supplemental Figure S3 Blots with molecular markers illustrated in Figure 8.

Figure 8B

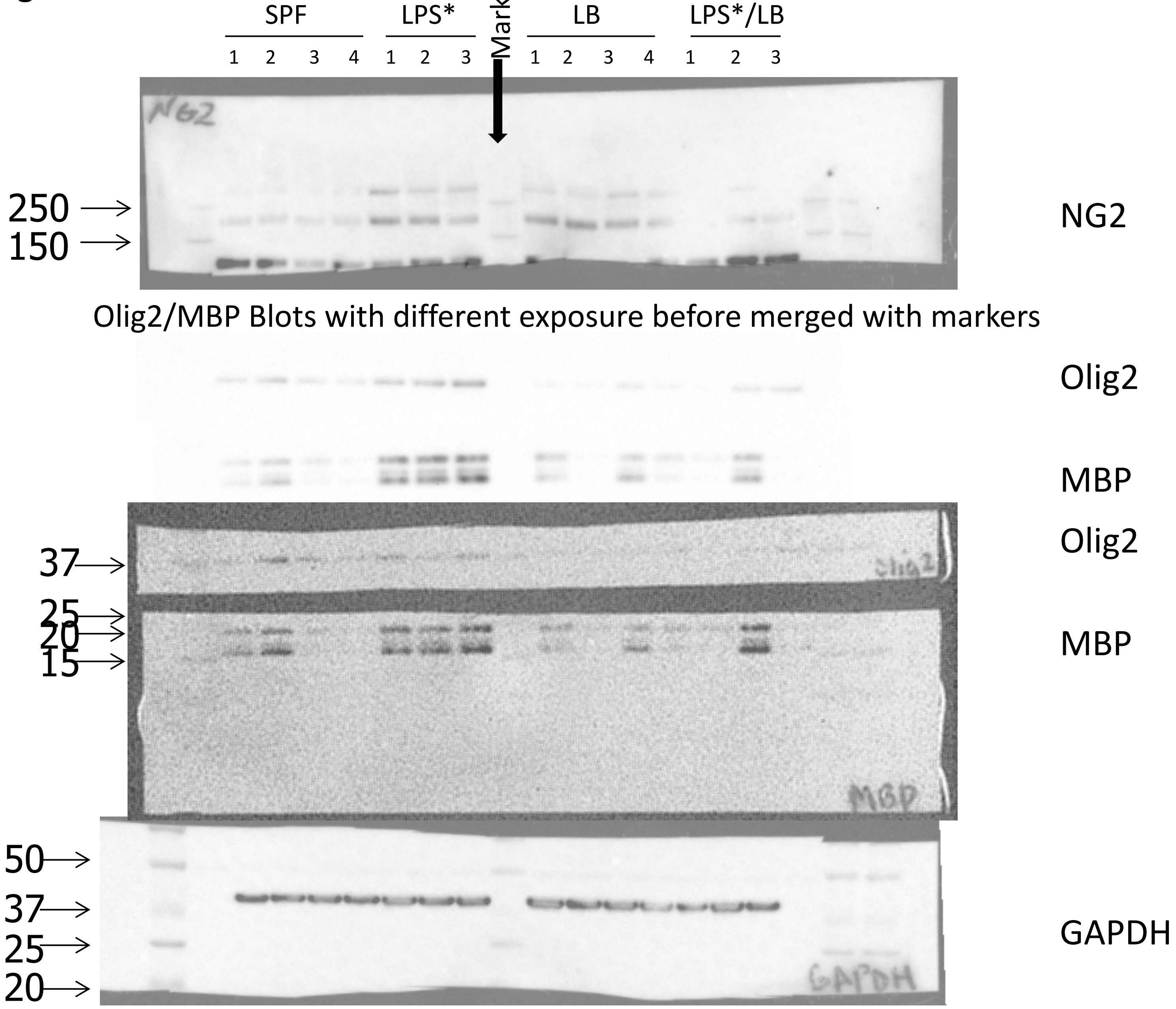

Supplement: Supplementary file 1 — Supplementary Information. [file 41598_2020_65180_MOESM1_ESM.pdf]
